# Characterizing fungal community shifts associated with *Amauromyza karli* Hendel (Diptera: Agromyzidae) infestation in quinoa

**DOI:** 10.3389/fpls.2026.1741091

**Published:** 2026-04-22

**Authors:** Neha Panwar, Jane E. Stewart, Jorge R. Ibarra Caballero, Adrianna Szczepaniec

**Affiliations:** Department of Agricultural Biology, Colorado State University, Fort Collins, CO, United States

**Keywords:** Chenopodium quinoa, fungal diversity, integrated pest management, microbiome, pest abundance

## Abstract

**Introduction:**

Fungal communities are central elements of phytobiomes, yet their roles in mediating plant-insect interactions remain poorly understood. Here, we addressed this knowledge gap in quinoa, which has recently suffered significant losses due to a stem-boring pest.

**Methods:**

We used culture-based isolation from stems and amplicon-based profiling of rhizosphere soils to characterize quinoa-associated fungi across six site-year combinations in Colorado and to relate community patterns to abundance of stem-boring fly *Amauromyza karli* Hendel (Diptera: Agromyzidae).

**Results:**

Eighteen stem endophytes dominated by Ascomycota were isolated. Soil sequencing resolved 23 core amplicon sequence variants detected across all site-years; the core was primarily Ascomycota, with *Fusarium* spp., *Alternaria* spp., and *Plectosphaerella* spp. comprising over half of relative abundance of the entire community. Alpha diversity (richness, Shannon, inverse Simpson) differed significantly among site-years, and beta-diversity analyses revealed clustering by site and year. Abundance of adult *A. karli* was correlated positively with soil fungal richness and Shannon diversity and was also significantly associated with differences in community composition. Indicator and differential-abundance analyses identified taxa linked to low fly abundance (e.g., *Cladosporium herbarum*, *Alternaria* spp.) versus high abundance (e.g., *Fusarium* solani, *Microdochium* spp.). *Fusarium* spp., and *Alternaria* spp. were more prevalent in fields with high larval abundance, whereas antagonistic endophytes such as *Gibellulopsis piscis* and *Heydenia* spp. dominated in low-abundance fields.

**Discussion:**

These results indicated that community composition impacted pest pressure, with pathogenic fungi coinciding with higher fly abundance and entomopathogenic fungi enriched where larval pressure was lower. These findings identify candidate taxa for microbiome-informed integrated pest management and underscore the potential of site-specific practices (e.g., intercrops, organic amendments) to foster fungal communities that enhance quinoa resilience.

## 1 Introduction

Plants coexist with diverse microbial communities known as microbiomes that can influence plant performance and stress tolerance. Fungi are central component of these communities because they can act as endophytes and are often closely associated with plant roots. These interactions can result in altered nutrient acquisition, affect plant physiology, and the expression of defense traits (Sen, 2005; Cregger et al., 2018; Terhonen et al., 2019; Pascale et al., 2020; Noman et al., 2021; Trivedi et al., 2022). Fungal communities have been shown to mediate resistance against insect herbivores as well. For example, Frew and Wilson (2021) reported that the diversity and composition of arbuscular mycorrhizal fungal communities significantly increased plant resistance to insect herbivory. In this study, wheat, *Triticum aestivum* L. (Poales: Poaceae) inoculated with a diverse native arbuscular mycorrhizal fungal community had 35% higher phenolic content, and the generalist herbivore, *Helicoverpa punctigera* Wallengren (Lepidoptera: Noctuidae) exhibited a 51% lower growth rate when feeding on these plants compared to plants lacking arbuscular mycorrhizal fungi. The mechanisms underlying these effects can be diverse and include improved host defenses through enhanced production of secondary metabolites, priming hormonal defense pathways such as jasmonic acid or salicylic acid, and enhancing plant vigor and nutritional balance (Coppola et al., 2019).

Despite growing interest in plant microbiomes, the fungal communities associated with crops and their role in crop protection remain understudied. Quinoa, *Chenopodium quinoa* Willd (Caryophyllales: Amaranthaceae), represents a crop for which associated fungal communities remain poorly characterized, yet these communities may prove useful in informing pest management strategies. Quinoa is grown worldwide and its adaptability to grow in marginal environments has led to expansion of its cultivation from quinoa’s native Andean region to North America, Europe, and Asia (Alandia et al., 2020). There is a growing interest in identifying the mechanisms underlying the resilience of this crop and how it interacts with the microbial communities to improve growth and stress resilience (González-Teuber et al., 2022; Hu et al., 2022; Maestro-Gaitán et al., 2025). While recent studies have started to explore the bacterial microbiome of quinoa (Hu et al., 2022; Maestro-Gaitán et al., 2025), research on fungal microbiome particularly endophytic fungi within stems and the soil fungal community remains limited.

Since quinoa is generally grown in areas with high incidence of abiotic stress such as drought, high salinity and extreme temperature fluctuations, these fungal associations may play an important role in mediating nutrient acquisition, stress tolerance, and improving defenses against herbivores. For example, root endophytic fungi such as *Penicillium murcianum* have been shown to improve quinoa’s salt tolerance by increasing the activities of defense enzymes such as phenyl ammonia-lyase by approximately 33% (González-Teuber et al., 2022). Beyond individual interactions, a whole-microbiome perspective is needed to advance the application of fungal-plant interactions for crop protection in quinoa. However, to our knowledge, no studies have examined how quinoa-associated fungal communities influence interactions with insect pests. This knowledge gap is particularly important as expansion of quinoa cultivation into new regions can increase pest pressure from the native herbivores such as *Amauromyza karli* Hendel (Diptera: Agromyzidae) in North America (Szczepaniec and Alnajjar, 2023). The larvae of this native fly feed within quinoa stems, disrupt the vascular system, and reduce water and nutrient uptake, which leads to wilting, lodging, and severe yield losses in quinoa (Szczepaniec and Alnajjar, 2023). Severe infestations often result in substantial yield losses, in some cases approaching 100%. For example, infestations by this pest were associated with a decline in quinoa acreage in San Luis valley region of Colorado from 3000 acres in 2021 to only 900 acres in 2022 (Szczepaniec and Alnajjar, 2023).

Despite the severity of these outbreaks, current integrated pest management strategies for quinoa lack a comprehensive understanding of the biotic factors influencing pest dynamics; in particular, the fungal community may represent a key missing component in our ability to predict and manage outbreaks of *A. karli*. While quinoa is highly susceptible to this pest, little is known about how its associated fungal microbiota may influence interactions between the crop and *A. karli*. Therefore, our objective was to characterize fungal communities associated with quinoa cultivation and assess whether fungal community structure can explain the abundance patterns of *A. karli.* If fungal associations benefit quinoa, selecting genotypes that promote associations with putative entomopathogenic fungi, or adopting soil management practices that promote a resilient microbial community can improve crop protection (Arif et al., 2020). This was illustrated by Enders et al. (2024) who used plant-guided selection over multiple generations to engineer rhizosphere microbiomes of tomato, *Solanum lycopersicum* L. (Solanales: Solanaceae) that reduced performance of potato aphid, *Macrosiphum euphorbiae* Thomas (Hemiptera: Aphididae) by up to 20% in some generations. Although these effects were transient, this study shows the potential for manipulating microbial communities to defend plants against herbivory. Therefore, understanding the composition and activity of fungal microorganisms in interaction with plants can aid in agricultural production aimed at enhancing long-term sustainability of quinoa.

This study aimed to (1) isolate and identify culturable endophytic fungi from quinoa stems, (2) characterize fungal species present in quinoa-cultivated soils using amplicon-based high-throughput sequencing across multiple sites and growing seasons and (3) explore correlations between *A. karli* abundance and fungal diversity. By characterizing the fungal taxa associated with quinoa, we can evaluate their functional roles and explore microbiome-informed strategies for enhancing crop resilience and sustainability.

## 2 Materials and methods

### 2.1 Sampling

Stem and soil samples were collected from quinoa fields during the 2023 and 2024 growing seasons in the San Luis Valley region of Colorado. Quinoa has been cultivated in the San Luis Valley of Colorado since the 1980s as its high elevation of 7, 500 feet and arid climate resembles the crop’s native environment in the Andes. Three quinoa fields were sampled in both years. Of these, one field was organically managed (sampled in both years), while the remaining fields were conventionally managed (**Table 1**). Samples were collected in July in both years (26 July 2023 and 17 July 2024) when the plants were in the vegetative stage. At each location, samples were randomly selected with a minimum spacing of at least 150 meters between sampling points. Fungal communities in quinoa stems and soil were characterized by collecting between eight and 16 plants and soil samples per field in 2023 (N = 32) and 35 plants and soil samples were collected in each field in 2024 (N = 105) (**Table 1**). Sampling intensity differed between the years because 2023 was the first year of study and served to establish field and laboratory workflows. Based on the initial results, sampling was extended in 2024 to increase replication and better capture within-field variability. Stem and rhizosphere samples were collected from different plants. The plants were clipped at the base using pruners for stem sampling, while three separate quinoa plants were randomly selected and rhizosphere soil adhering to the roots was collected for soil samples (Estrada et al., 2023). Soil from the three plants at each point was composited into one representative sample and transferred to Falcon™ 50 mL conical centrifuge tubes (Thermo Fisher Scientific, Massachusetts, USA). All samples were transported in coolers with ice bags to the laboratory at Colorado State University for further processing. Stem samples were processed the next day for endophytic fungi isolation, and soil samples were stored at -80 °C until DNA extraction and further analysis.

**Table 1:** Site characteristics, sampling intensity, and management practices for quinoa fields sampled for rhizosphere soil and stem endophytes in Colorado in 2023 and 2024.

Sampling year	Site code	Latitude (°N)	Longitude (°W)	Field size (in acres)	County	Number of stem samples	Number of soil samples	Management type
2023	Alamosa	37°37′42.1″	105°53′30.3″	120	Alamosa	16	16	Organic
	Rio_1	37°42′34.8″	106°07′59.8″	0.5	Rio Grande	8	8	Conventional
	Yuma	40°16′11.6″	102°46′24.9″	60	Yuma	8	8	Conventional
2024	Alamosa	37°38′11.9″	105°54′56.3″	60	Alamosa	35	35	Organic
	Rio_2	37°42′45.9″	106°08′05.3″	5	Rio Grande	35	35	Conventional
	Rio_3	37°42′28.3″	106°06′34.9″	60	Rio Grande	35	35	Conventional

Site code refers to unique field identifiers; sites with the same prefix (e.g., Rio_1, Rio_2, Rio_3) are distinct fields within the same county.

Stem and rhizosphere soil samples were collected in equal numbers within each site-year; however, samples were not paired (i.e., stem and soil samples originated from different plants or sampling points).

### 2.2 Isolation and identification of stem endophytes

Stem tissues approximately 10 cm above the basal portion of the plants were used for isolation of endophytic fungi. These samples were plated on three different media including potato dextrose agar media (9 g of agar, 18 g of potato dextrose broth, and 1 L of distilled water), V8 media (200 mL of V8 juice, 2 g of CaCO_3,_ 15_ g_ of agar, and 1 L of distilled water) and yeast mannitol agar media (1 g of yeast extract, 10 g of mannitol, 15 g of agar, 0.5 g of K_2_HPO_4_, 0.2 g of MgSO_4_, 0.1 g of NaCl, 0.1 g of CaCO_3_ and 1 L of distilled water). Prior to plating, stem samples were surface-sterilized by three successive applications of 70% ethanol, each application followed by flaming. This method was chosen to avoid ethanol from penetrating the internal tissues and killing the endophytes. To ensure that only internal tissues were used for fungal isolation, the sterilized ends of each stem segment were trimmed and discarded using a sterile scalpel. The remaining segments were then aseptically dissected, and the interior surface of the samples were placed in direct contact with the agar surface. Plates were incubated at 23 °C and regularly monitored for fungal growth. Sterilization efficacy was visually confirmed during incubation; only fungal mycelia emerging directly from the internal tissue were sub-cultured onto new plates, while any growth originating from the exterior stem surface was considered as surface contamination and discarded. These isolates were then grown to obtain pure cultures for further DNA extraction.

To obtain mycelia for DNA extraction, agar plugs that were approximately 5 mm in diameter were taken from the actively growing edge of the mycelia and were transferred to potato dextrose broth. The cultures were grown for about 2 weeks on an Innova 2300 rotary shaker (New Brunswick Scientific, New Jersey, USA). These cultures were then vacuum filtrated using a Fisherband vacuum pump (Model G180GDX, Fisher Scientific, Pennsylvania, USA), i.e., passed through Whatman filter paper under reduced pressure to rapidly remove excess surface broth and harvested mycelia was stored at -80 °C for further DNA extraction.

DNA was extracted from approximately 50 mg of fungal tissue using the SYNERGY™ 2.0 Plant DNA Extraction Kit (OPS Diagnostics, New Jersey, USA), following the manufacturer’s instructions. Samples were homogenized in a plant homogenization buffer using a FastPrep-24 bead beater (MP Biomedicals, California, USA). DNA was precipitated with cold isopropanol and then bound to silica spin columns, washed with cold 70% ethanol, and eluted in TE buffer (pH 8). The quality of the DNA was assessed using Nanodrop-1000 spectrophotometer (Thermo Fisher Scientific, Massachusetts, USA). The internal transcribed spacer (ITS) region was amplified and sequenced using primers ITS 1 (5′-GGAAGTAAAAGTCGTAACAAGG-3′) and ITS 4 (5′-TCCTCCGCTTATTGATATGC-3′) whereas the translation elongation factor 1-α (EF1-α) region was amplified and sequenced using primers EF983F (5′- GCYCCYGGHCAYCGTGAYTTYAT-3′) and 1567R (5′-ACHGTRCCRATACCACCSATCTT-3′) (**Supplementary Table S1**). We sequenced two loci (ITS and EF1-α) to increase confidence in isolate identification. ITS was used for broad fungal barcode identification, whereas EF1-α provided additional phylogenetic resolution to distinguish closely related taxa and to confirm identifications when ITS matches were ambiguous. The polymerase chain reaction (PCR) was performed using a T100 thermal cycler (Bio-Rad Laboratories, California, USA). The PCR conditions included an initial denaturation at 94 °C for 1 minute, followed by 40 cycles of 95 °C for 30 seconds, 60 °C for 30 seconds, and 72 °C for 45 seconds, with a final extension at 72 °C for 10 minutes.

PCR amplicons were visualized via gel electrophoresis using a VWR horizontal electrophoresis system (Model E1007-10, VWR International, Radnor, PA, USA) powered by a PowerPac 300 power supply (Bio-Rad Laboratories, California, USA), cleaned using ExoSAP-IT PCR Product Cleanup Reagent (Thermo Fisher Scientific, California, USA), and submitted to Eurofins Genomics (Louisville, Kentucky, USA) for Sanger sequencing using the previously mentioned primers. The sequences were aligned using CLC Main Workbench 24 and the resulting consensus sequences were then identified by performing a BLASTn search against the NCBI database (https://blast.ncbi.nlm.nih.gov/Blast.cgi) to determine fungal species.

### 2.3 Soil DNA extraction and sequence processing

Approximately 250 mg of soil was used for DNA extraction using the DNeasy PowerSoil Pro Kit (Qiagen, California, USA), following the manufacturer’s instructions. Samples were bead beaten for 10 min in PowerBead Pro tubes with lysis buffer (CD1), clarified with CD2, mixed with binding buffer (CD3), passed over silica spin columns, washed (EA, C5), and eluted in 50 µl of C6 (10 mM Tris, pH 8). DNA quantity and quality were evaluated using a Nanodrop 1000 spectrophotometer (Thermo Fisher Scientific, Massachusetts, USA) and approximately 50 µL of each sample was submitted to Novogene Corp. Inc. (California, USA) for amplicon-based metabarcoding. The fungal ITS2 region was amplified using ITS3 (5′-GCATCGATGAAGAACGCAGC-3′) and ITS4 (5′-TCCTCCGCTTATTGATATGC-3′).

### 2.4 Soil microbiome analysis

Raw ITS2 reads were assessed with FastQC (Andrews, 2010). All libraries showed high per-base quality (median ≥Q30 across read lengths) and no detectable adapter contamination; therefore, no adapter or quality trimming was performed prior to downstream analyses. All subsequent analyses were conducted in R (v.4.4.1; R Core Team, 2024). The dada2 package (v.1.36.0; Callahan et al., 2016) was used to generate an amplicon sequence variant (ASV) table for fungal communities. Taxonomic classification of ITS2 ASVs was performed by aligning sequences against the UNITE (Abarenkov et al., 2024) database. ITS2 sequencing also captured ASVs from non-fungal eukaryotes, which were excluded from downstream analyses.

Twenty samples were removed from analysis because of the low number of sequencing reads (<10, 000 reads per sample). Rarefaction curves were generated using the phyloseq package (v.1.52.0; McMurdie and Holmes, 2013) in R (v.4.4.1; R Core Team, 2024) to evaluate sequencing depth. To visualize fungal community composition, ASVs were agglomerated at the genus level using phyloseq package (v.1.52.0; McMurdie and Holmes, 2013). Stacked bar plots were generated using the ggplot2 package (v.4.0.2; Wickham, 2016) to display dominant genera across fields and sampling years. Core fungal taxa were identified based on presence in more than 50% samples from each field site-year combination. The resulting core ASVs were used to subset the normalized abundance table, and only taxa with relative abundance >1% in at least one sample were retained for visualization. A heatmap was generated using the pheatmap package (v.1.0.13; Kolde, 2025) in R (v.4.4.1; R Core Team, 2024).

Alpha diversity was assessed using richness, Shannon’s diversity index (*H*′) and the inverse Simpson diversity index (1/*D*), all calculated with phyloseq package (v.1.52.0; McMurdie and Holmes, 2013). Shannon’s index emphasizes richness, while the inverse Simpson index gives more weight to evenness (Nagendra, 2002). To test differences in fungal diversity across field site-year combinations, Type III analysis of variance (ANOVA) was conducted using the car package (v.3.1.5; Fox and Weisberg, 2019). ANOVA assumptions were evaluated using Shapiro-Wilk tests for normality and Levene’s test for homogeneity of variance. Data transformations were applied where necessary. Where significant effects were found (P < 0.05), Tukey-adjusted pairwise comparisons were conducted.

Compositional differences between fungal communities were visualized using ordination plots. Data were normalized using cumulative sum scaling prior to analysis. The plots were generated using non-metric multidimensional scaling (NMDS) and principal coordinate analysis (PCoA) in the phyloseq and vegan (Oksanen et al., 2022) packages. To ensure that the unbalanced sampling design did not bias beta diversity comparisons, a subsampling-based sensitivity analysis was performed. The larger 2024 samples were randomly downsampled over 100 iterations to match the minimum sampling intensity (N = 8) of the 2023 fields. Permutational multivariate analysis of variance (PERMANOVA) was performed for each iteration to verify the stability of the community patterns. PERMANOVA was performed in R (v.4.4.1; R Core Team, 2024) to test for differences in fungal community structure among field site-year combinations. Additionally, homogeneity of multivariate dispersion was confirmed using the betadisper function in the vegan package (v.2.7.2; Oksanen et al., 2022). A separate PERMANOVA was performed to test for differences in fungal community structure among management type as well.

To compare cultured stem endophytes with soil metabarcoding data, overlap was quantified at genus and species levels. Since the soil dataset targeted ITS2 while stem isolate sequences spanned the full ITS region, species-level concordance was evaluated conservatively by aligning representative soil ITS2 ASV sequence to the corresponding stem isolate ITS consensus using BLASTn (bl2seq). A minimum threshold of 95% sequence identity was used to assess sequence concordance for taxa detected in both datasets.

### 2.5 Abundance of adults and larvae of *A. karli*

Adult activity of the *A. karli* was monitored with yellow sticky traps (12.7 cm x 17.78 cm, gridded, ARBICO Organics^®^ Yellow Insect Traps, AZ, USA). Cards were mounted on bamboo sticks with metal clips and were placed at least 100 m apart within each field to monitor the adult fly activity. Six traps were placed in each field in 2023, and five traps were deployed per field in 2024. Traps were installed within 15 days of sowing, *i.e.*, between 24 May and 13 June in 2023, and between 28 April and 11 June in 2024, and were replaced at weekly intervals until captures declined to zero across all the sites. Traps were wrapped in clear plastic wrap to prevent adhesion, and transported in coolers with ice packs to the laboratory at Colorado State University and stored at 4 °C. The traps were inspected using a dissecting microscope (Jenco DG6-2L 7-45X Stereo Zoom Binocular Microscope, Jenco International, Portland, OR 97222) to assess the number of adult *A. karli*. The adults were identified using their key characteristics, *i.e.*, yellow head and yellow joints between femur and tibia (Lonsdale, 2021). Counts from all traps within a field were averaged for each sampling date to obtain a field-level daily mean, and these date-specific means were then averaged across the season to obtain the seasonal adult abundance.

Larval densities were quantified in 2024. At each location, fifty plants were destructively sampled at biweekly intervals. Sampling began on 4 June and ended on 13 August. Each of the three fields was divided into five sections, from which ten plants were selected, spaced at least 100 m apart (N = 50 for each field and each sampling date). Plants were clipped at the base with pruners, placed in labeled bags, transported on ice to the laboratory at Colorado State University, and held at 4 °C overnight until processing the next day. In the laboratory, stems were split longitudinally with a scalpel, and larvae per stem were counted; stem length and diameter were also recorded. For each field and sampling date, larval density (number of larvae per cm^3^) was computed as the mean across the 50 plants, and date-level means were averaged across the season to obtain the seasonal larval abundance. Larvae collected from quinoa stems were submitted to C.P. Gillette Museum of Arthropod Diversity, Colorado State University, Colorado, USA for formal identification by a taxonomist. Specimens were identified using morphological diagnostic characteristics and voucher specimens were deposited in the museum.

### 2.6 Microbial community analysis in relation to insect abundance

To investigate relationships between insect abundance and microbial diversity, we calculated Spearman correlation coefficients between the abundance of *A. karli* and alpha diversity indices (Richness, Shannon, and inverse Simpson). Furthermore, to explore the correlation between community composition and *A. karli* abundance, envfit function from the vegan package (v.2.7.2; Oksanen et al., 2022) was used to fit the insect abundance vector onto ordination space derived from NMDS based on Bray-Curtis dissimilarity. For community composition analysis, *A. karli* abundance was converted into binary categories (High vs. Low) based on median values (Aghdam et al., 2024; Olson et al., 2024), and indicator species analysis was conducted using the indicspecies package (v.1.8.0; De Cáceres, 2013) on the core fungal dataset to identify core fungal taxa associated with each group.

Since larval abundance was quantified only in 2024; therefore, subsequent analyses linking larval abundance with soil fungal communities were conducted using the 2024 dataset. For larval abundance, the three fields were assigned based on median value into two categories – high and low abundance of *A. karli* (**Table 2**). To test for taxon shifts between larval‐abundance categories, differentially abundant ASVs were identified using Wald tests implemented in DESeq2 package (v.1.48.2; Love et al., 2014), with Benjamini-Hochberg correction applied to control the false discovery rate (Benjamini and Hochberg, 1995). Effect sizes were reported as log_2_ fold change. A heatmap was constructed using pheatmap function to visualize the difference between groups. As stem endophytic fungi were identified using a culture-dependent approach and sanger sequencing which provides isolate identities rather than quantitative abundance table, microbial community analysis could only be conducted for the soil fungal ASV dataset.

**Table 2:** Adult trap captures and larval densities of *Amauromyza karli* across site-years, with classification into relative density categories.

Sampling year	Site code	Adult densities per trap	Adult relative density category	Larval densities per cm^3^ stem tissue	Larval relative density category
2023	Alamosa	0.212	Low	NA	NA
	Rio_1	0.456	High		
	Yuma	1.648	High		
2024	Alamosa	0.186	Low	0.511	High
	Rio_2	0.320	High	0.159	High
	Rio_3	0.060	Low	0.001	Low

Density categories (High, Low) were assigned relative to the median across all site-years (Adults: 0.266 per trap; Larvae: 0.159 per cm³). Values ≥ median were classified as High; values < median as Low. Adult and larval densities were derived from independent sampling efforts and were not paired. *NA* indicates that larval sampling was not conducted for the site-year.

## 3 Results

### 3.1 Characterization of fungal endophytes from quinoa stems and soil rhizosphere

We recovered 18 isolates from quinoa stems (**Table 3**). The majority of these isolates belonged to the phylum Ascomycota. Pleosporales was the most dominant order followed by Glomerellales and Hypocreales. Three isolates (*Epicoccum layuense, Penicillium citreonigrum*, *Aspergillus flavus*) lacked EF1-α sequences because of poor sequencing quality. The most frequent taxa recovered from stems was *Alternaria alternata* followed by *Fusarium equiseti*.

**Table 3:** Taxonomic identification and sequence similarity of fungal endophytes isolated from quinoa stems based on ITS and EF1-α loci.

Year of isolation	Phylum	Order	Description	Closest GenBank accession			
				ITS accession	ITS identity	EF1-α accession	EF1-α identity
2023	Ascomycota	Pleosporales	*Alternaria alternata*	ON208242.1	99.82%	PV388123.1	99.75%
2023	Ascomycota	Hypocreales	*Fusarium equiseti*	MK168567.1	99.80%	KU939018.1	99.77%
2023	Ascomycota	Pleosporales	*Alternaria alternata*	ON712034.1	99.82%	ON993381.1	99.77%
2023	Ascomycota	Hypocreales	*Fusarium equiseti*	ON500604.1	99.80%	KU939018.1	99.76%
2023	Ascomycota	Hypocreales	*Fusarium equiseti*	MK226296.1	99.17%	KU939018.1	100%
2023	Ascomycota	Glomerellales	*Plectosphaerella cucumerina*	MW850542.1	100%	LR026503.1	100%
2023	Ascomycota	Pleosporales	*Epicoccum layuense*	MT626579.1	99.79%	NA	NA
2023	Mucoromycota	Mortierellales	*Mortierella alpina*	KJ469807.1	100%	MN878037.1	99.09%
2023	Ascomycota	Glomerellales	*Gibellulopsis serrae*	KY611819.1	99.61%	LR026432.1	100%
2023	Ascomycota	Glomerellales	*Gibellulopsis nigrescens*	KJ534578.1	100%	LR026431.1	100%
2023	Ascomycota	Hypocreales	*Cosmospora lavitskiae*	KU563624.1	100%	OQ470831.1	98.96%
2023	Ascomycota	Glomerellales	*Gibellulopsis nigrescens*	AB551216.1	100%	LR026431.1	100%
2024	Ascomycota	Eurotiales	*Penicillium citreonigrum*	MH857876.1	100%	NA	NA
2024	Ascomycota	Glomerellales	*Plectosphaerella cucumerina*	OW985795.1	100%	LC633928.1	100%
2024	Ascomycota	Eurotiales	*Aspergillus flavus*	MN547373.1	99.82%	NA	NA
2024	Mucoromycota	Mortierellales	*Mortierella alpina*	KJ469807.1	100%	MN878037.1	99.31%
2024	Ascomycota	Pleosporales	*Alternaria alternata*	MT446174.1	100%	PV590953.1	99.77%
2024	Ascomycota	Pleosporales	*Alternaria alternata*	OR826587.1	100%	PV590953.1	100%

Taxonomic assignments were based on BLASTn searches against the NCBI GenBank database using ITS and EF1-α sequences, and percent identity represents sequence similarity to the closest GenBank match. EF1-α sequences were not obtained for three isolates; these are indicated as NA.

ITS, internal transcribed spacer region; EF1-α, translation elongation factor 1-alpha.

There was substantial overlap between stem isolates and soil ASVs at the genus level (66.7%) but more limited overlap at the species level (30%). Of the ten species isolated from stems, three species including *A. alternata, F. equiseti* and *Plectospharella cucumerina* occurred in both stems and soils. Since metabarcoding targeted only ITS2 region whereas cultured isolates were sequenced across the full ITS, we quantified sequence concordance by aligning each ITS2 ASV to its corresponding full-length ITS using NCBI BLASTN (bl2seq); percent identities were 99.64% for *A. alternata*, 99.61% for *F. equiseti*, and 95.24% for *P. cucumerina*. Six out of the total nine genera were shared between stems isolates and soil ASVs. These included *Alternaria* spp., *Fusarium* spp., *Plectospharella* spp., *Mortierella* spp., *Gibellulopsis* spp., and *Penicillium* spp. Three species including *A. flavus*, *E. layuense* and *Cosmospora lavitskiae* were detected only from stem samples. However, these overlaps should be interpreted conservatively due to the fundamental differences in methodology between the two datasets. Our comparison relied on culture-based identifications for stem isolates (a method known for its low throughput and inherent bias toward fast-growing, culturable Ascomycota) whereas the soil core was analyzed via amplicon metabarcoding. Consequently, the limited overlap observed likely reflects differences in detection sensitivity and taxonomic resolution resulting from the use of different ITS target regions. Future studies should employ culture-independent validation, such as high-throughput sequencing of stem tissues, to provide a more comprehensive and unbiased comparison of the fungal communities across these two niches.

**Figure 1:**
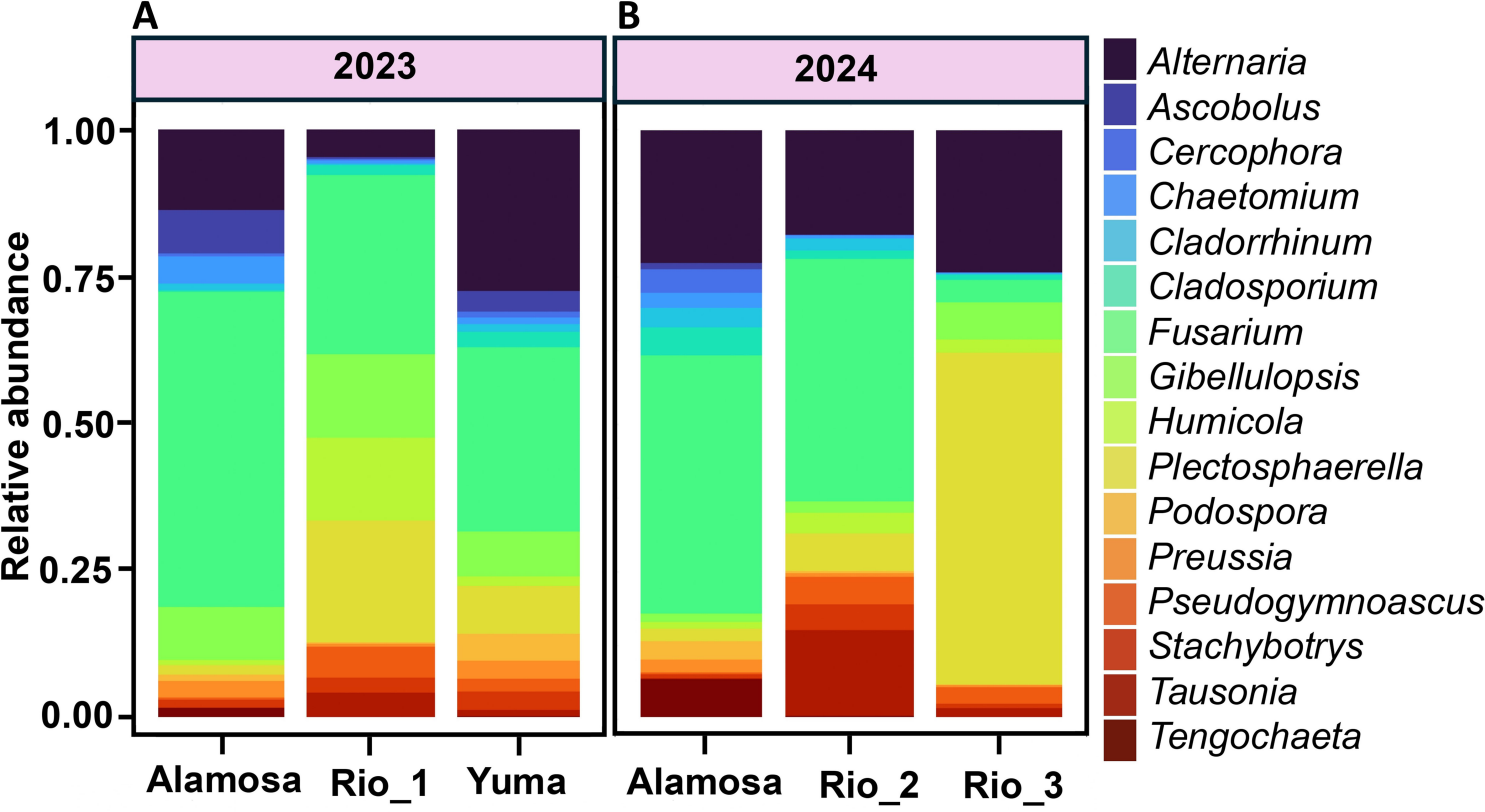
Relative abundance of dominant fungal genera (≥1% mean abundance) across quinoa fields in 2023 **(A)** and 2024 **(B)**. Stacked bars display the mean relative abundance of the 16 most abundant genera (denoted by different colors) across different fields and year. Each bar represents the average across all samples from that field-year combination. *Fusarium*, *Alternaria* and *Plectosphaerella* together contributed more than half of the relative abundance across all the field-year combinations, but their proportions shifted across fields and year.

We detected 16 fungal genera with a mean relative abundance ≥1% in at least one of the six site-year combinations from the soil microbiome (**Figure 1**). Relative abundance profiles indicated differences in the fungal composition across sites as well as years. *Fusarium, Alternaria*, and *Plectosphaerella* collectively accounted for over 50% of total relative abundance across all site-year combinations, making them the most dominant genera and were most abundant in our stem samples. Among these, *Fusarium* was consistently abundant across all fields, except at Rio_3_2024, where *Plectosphaerella* was the most abundant.

**Figure 2:**
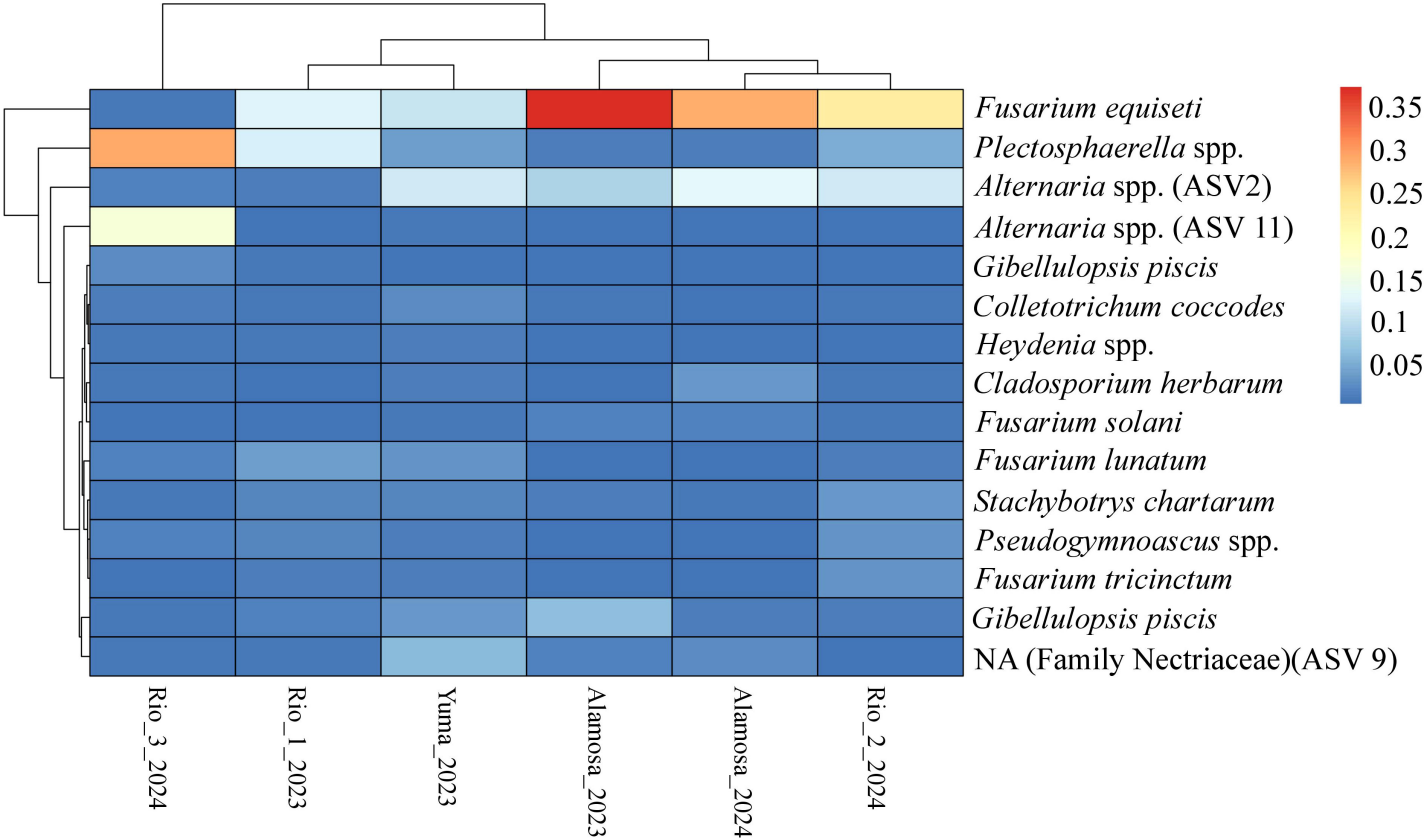
Heatmap of core fungal amplicon sequence variants (ASVs) at genus level across field site-year combinations in Colorado quinoa fields. The figure displays the mean relative abundance of core ASVs (ASVs present in at least 50% samples from each field site-year) across six field site-year combinations. Both rows (ASVs) and columns (site-year groups) are hierarchically clustered based on abundance profiles. Relative abundances were averaged across all samples within each field site-year. The color scale indicates the mean relative abundance (blue = low, red = high).

The core mycobiome was defined as ASVs detected in at least 50% samples from each of the six site-year combinations, resulting in 23 core ASVs (**Supplementary Table S2**). Five ASVs including *Alternaria* spp., *F. equiseti, Gibellulopsis piscis, Plectosphaerella* spp., and *Stachybotrys chartarum* were identified in all 117 samples. The core was dominated by Ascomycota (22 ASVs) followed by Mortierellomycota (one ASV). FUNGuild assigned ecological roles to 23 ASVs, with 17 ASVs categorized as plant pathogens, frequently coupled with endophytic and/or saprotrophic roles (Nguyen et al., 2016). Based on trophic mode, 13 ASVs were classified as pathotroph-saprotroph-symbiotroph, followed by pathotroph-saprotrophs (three ASVs). The relative abundances of these core ASVs shifted markedly between fields as well as years (**Figure 2**). Several core genera, including *Fusarium, Alternaria, Cladosporium*, and *Plectosphaerella*, dominated the community but showed substantial variability among sites and years. For instance, *Fusarium* (ASV1) reached peak abundance in Alamosa_2024, with multiple *Fusarium* ASVs abundant across several site-years, suggesting a stable presence of this genus. In contrast, *Alternaria* (ASV2, ASV8, ASV11) demonstrated distinct peaks in specific site-years.

### 3.2 Alpha diversity

**Figure 3:**
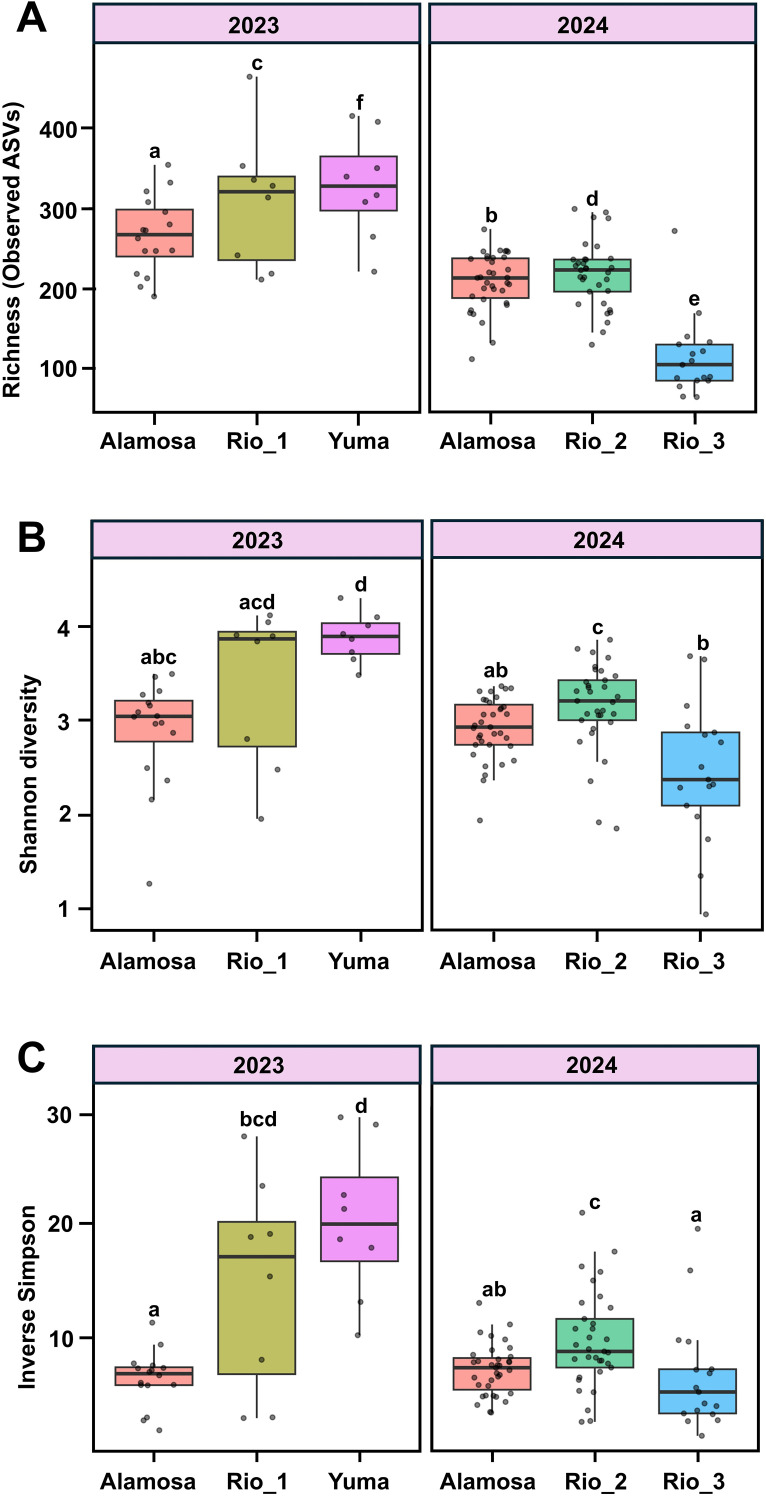
Alpha indices of fungal communities associated with quinoa rhizosphere soils across different field sites in 2023 and 2024. Boxplots show **(A)** Observed richness (number of ASVs), **(B)** Shannon diversity, and **(C)** inverse Simpson diversity index for each field site, grouped by year. Boxes represent the interquartile range for each field site-year combination, with the horizontal line indicating the median. Whiskers show the range excluding outliers, and each points represents an individual sample. Significant differences across field-year combinations were found for observed richness (Type III ANOVA, F = 32.80, df = 5, 111, P < 0.001), Shannon diversity (Kruskal-Wallis test, χ² = 36.3, df = 5, P < 0.001) and inverse Simpson index (Kruskal-Wallis test, χ² = 34.3, df = 5, P < 0.001). Different letters mean they were significantly different from each other (P < 0.05).

Alpha diversity, measured as richness, Shannon, and inverse Simpson indices varied significantly among site-year combinations, reflecting differences in both the number of fungal taxa and their evenness of distribution. The number of fungal taxa (richness) significantly differed across field site-year groups (F = 32.80, df = 5, 111, P < 0.001; **Figure 3A**), with Yuma_2023 exhibiting the highest richness and Rio_1_2024 the lowest. Shannon diversity also varied significantly among field-year combinations (Kruskal-Wallis test, χ² = 36.3, df = 5, P < 0.001; **Figure 3B**), with Yuma_2023 having highest Shannon index, indicating not only more taxa but also a more even distribution of their abundance. Similarly, there were significant differences among inverse Simpson diversity index for field-year combinations (Kruskal-Wallis test, χ² = 34.3, df = 5, P < 0.001; **Figure 3C**) with Yuma_2023 again having the highest diversity. This suggests that Yuma_2023 fungal communities were less dominated by a few abundant taxa, whereas other sites had stronger dominance by single or few groups.

### 3.3 Beta diversity

Beta diversity analyses revealed significant differences in fungal community composition among site-year combinations. Dispersion (variance) of community composition among groups did not differ significantly (F = 0.79, df = 5, 111, P = 0.56), confirming homogeneity of multivariate dispersions. On the other hand, fungal community composition differed significantly based on site-year groupings (F = 19.84, df = 5, 111, P = 0.001), with field site explaining 47% of the variance. Because PERMANOVA can be sensitive to unequal group sizes, we conducted a subsampling-based sensitivity analysis in which the original dataset was repeatedly resampled to equalize sample size across groups (n = 8 per group), and PERMANOVA was rerun for 100 iterations. The group effect remained significant in all iterations (P = 0.001), with R² values ranging from 0.468 to 0.592, indicating that the observed differences in fungal community structure were robust to sample-size imbalance. Management type was also significantly associated with fungal community composition when analyzed alone (PERMANOVA, R² = 0.21, P = 0.001). However, because management was confounded with site-year, its independent contribution could not be estimated in models that also included site-year.

**Figure 4:**
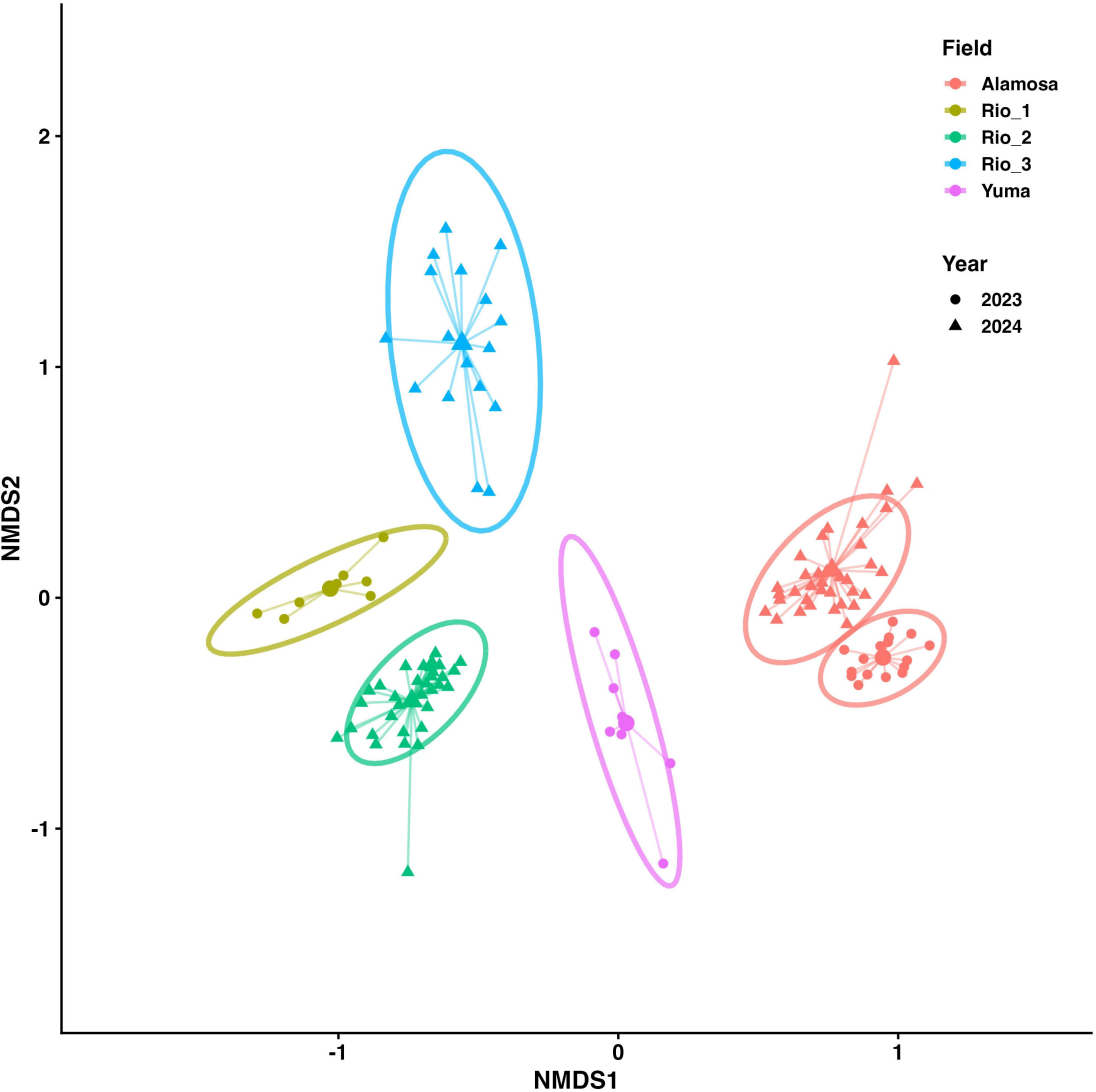
Non-metric multidimensional scaling (NMDS) of fungal community composition in quinoa rhizosphere soils across different field site-year combinations. Ordination was based on Bray-Curtis dissimilarity. Points represent individual samples, with colors indicating field sites and shapes representing sampling year. Centroid-linked spider diagrams and 95% confidence ellipses are overlaid to visualize clustering and dispersion among field-year combinations. Fungal communities clustered distinctly by site and year, suggesting strong spatial and temporal structuring of rhizosphere microbiota (PERMANOVA, F = 19.84, df = 5, 111, R^2^ = 0.47, P = 0.001).

**Figure 5:**
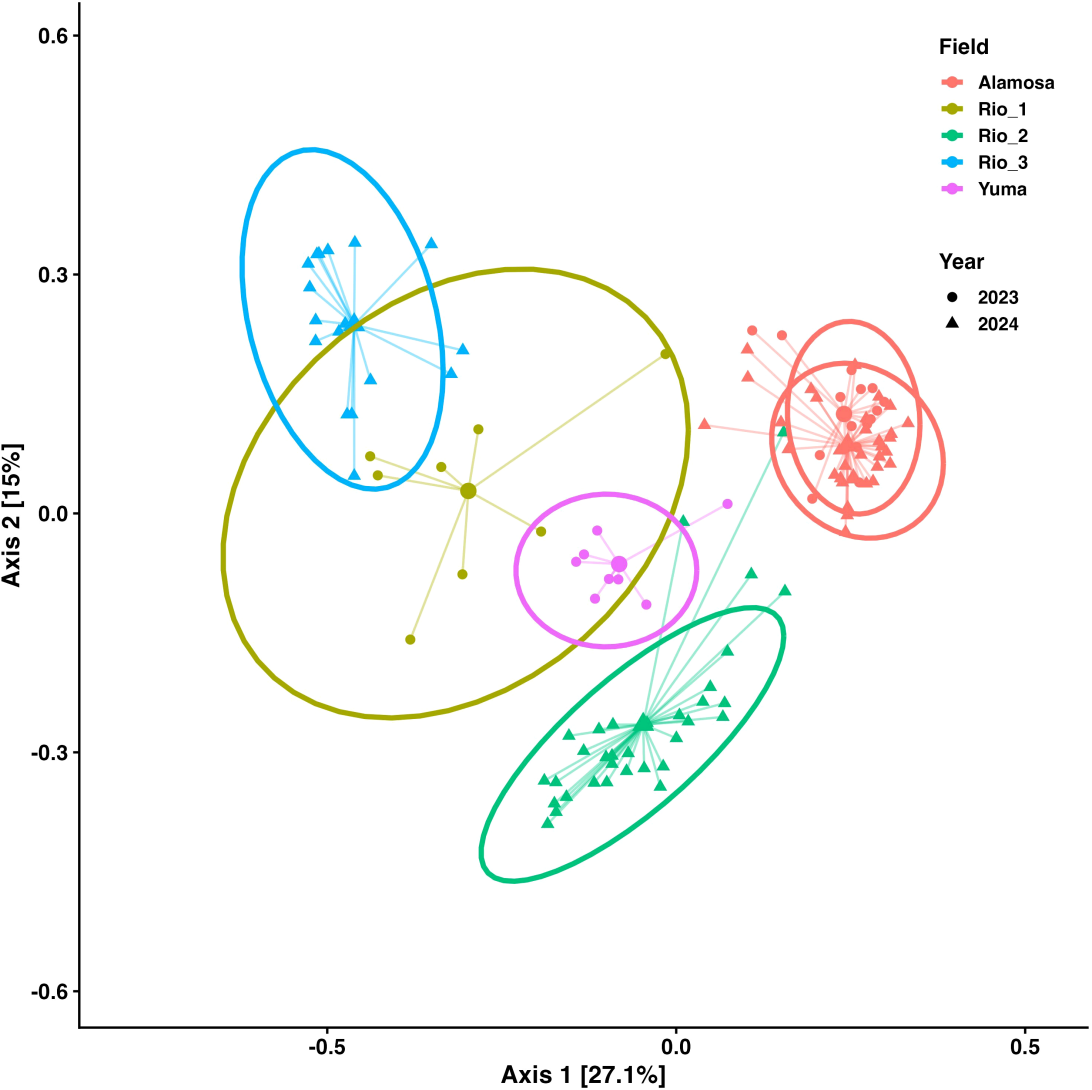
Principal Coordinates Analysis (PCoA) of fungal community composition in quinoa rhizosphere soils based on Bray-Curtis dissimilarity. Points represent individual samples, with colors indicating field sites and shapes representing sampling year. Centroid-linked spider diagrams and 95% confidence ellipses are overlaid to highlight patterns of clustering and dispersion among field-year combinations. Axis 1 and Axis 2 explain 27.1% and 15% of the total variation, respectively. Community structure differed significantly across field-year combinations (PERMANOVA, F = 19.84, df = 5, 111, R^2^ = 0.47, P = 0.001).

In the NMDS ordination plot (**Figure 4**), fungal communities clustered strongly by site and year with little overlap among groups. Alamosa_2023 and Alamosa_2024 grouped closely, suggesting temporal stability at this site. Yuma_2023 formed a distinct cluster, highlighting unique assemblages at this location. The PCoA plot explained 27.1% of variation on the first axis and 15% on the second (**Figure 5**). Similar to the NMDS, PCoA distinguished fungal assemblages by site and year. Alamosa communities clustered together across years, while Rio sites were more dispersed, indicating greater variability among fields within that region. Yuma_2023 formed a distinct cluster with no overlap, consistent with its higher alpha diversity. Together, the ordinations demonstrate strong spatial and temporal structuring of fungal communities, with some sites showing stability across years while others exhibited greater variability.

### 3.4 Correlation with *A. karli* abundance

Both Shannon diversity and observed richness positively correlated with adult *A. karli* abundance (n =6, ρ = 0.94, P = 0.017 for both indices). The 95% confidence intervals for these correlations (CI: 0.56-0.99) further support the reliability of this relationship, suggesting that sites with higher fungal diversity supported greater abundances of the adults of the stem-boring fly. The inverse Simpson diversity index also showed a positive but non-significant correlation (n = 6, ρ = 0.83, P = 0.058). The wider 95% confidence interval for this index (CI: 0.05-0.98) suggests a weak trend toward higher fungal diversity being associated with increased adult abundance. Furthermore, adult insect abundance was strongly associated with fungal community composition (R² = 0.2, P = 0.001). Out of the 23 core ASVs, 14 were significantly associated with variation in adult *A. karli* abundance (p < 0.05; **Supplementary Table S3**). Of these, 11 were associated with high *A. karli* abundance and 3 were associated with low abundance. Indicator ASVs significantly associated with low *A. karli* abundance included *Cladosporium herbarum*, *Alternaria* spp., and *Fusarium equiseti*, whereas taxa with high *A. karli* abundance included *Fusarium lunatum, Fusarium solani*, and *Fusicolla septimanifiniscientiae*.

**Figure 6:**
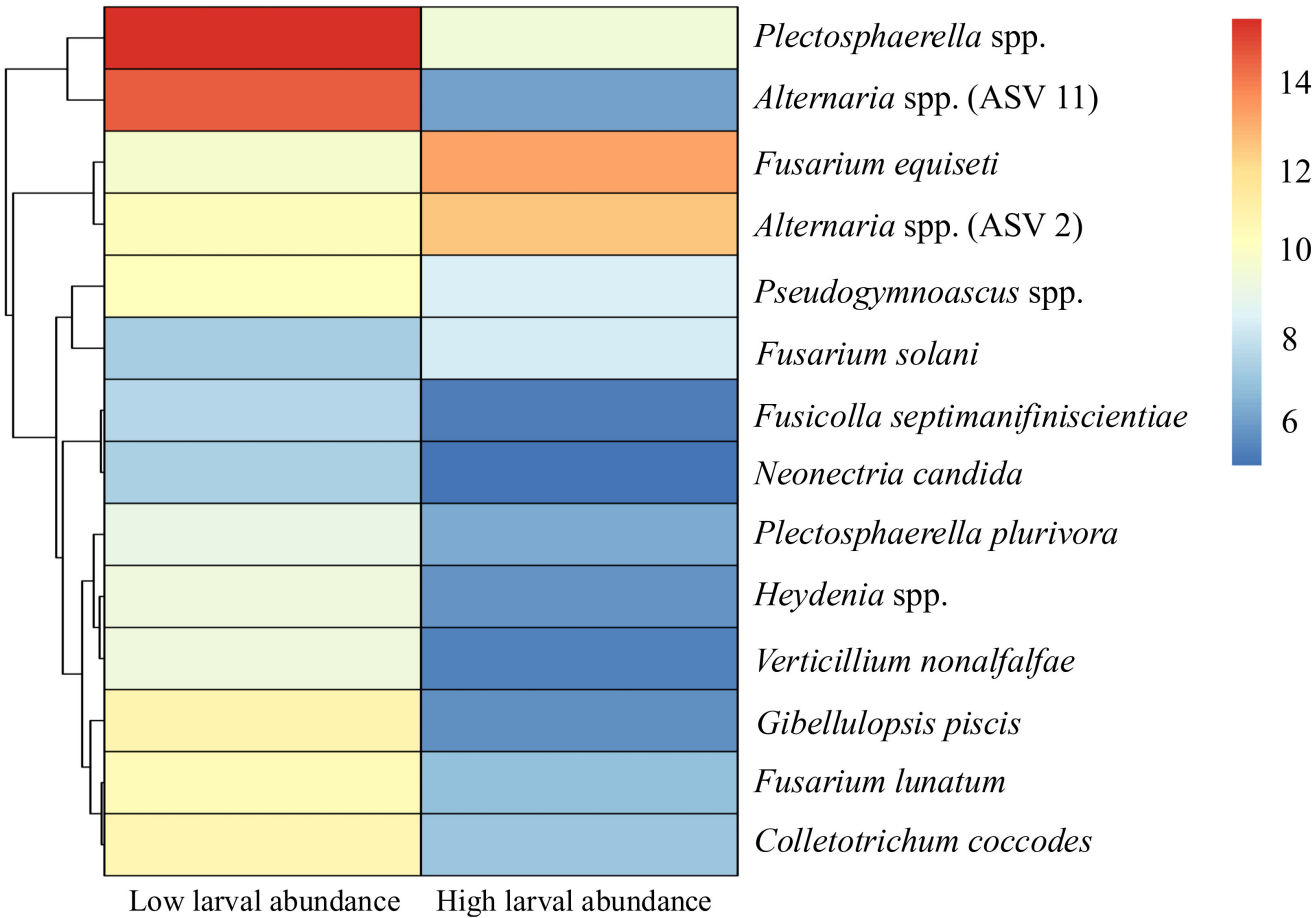
Differentially abundance of fungal taxa between fields with high and low *A. karli* larval abundance. Heatmap displays mean normalized counts, with red indicating higher abundance and blue indicating lower abundance. Rows represent specific fungal species. Significance was determined using Wald tests in DESeq2 with Benjamini-Hochberg correction (adjusted P < 0.05). Most of the taxa were more abundant in fields with low larval abundance, whereas *Fusarium equiseti* and *Alternaria* spp. (ASV2) were more abundant in high-larval fields.

**Figure 7:**
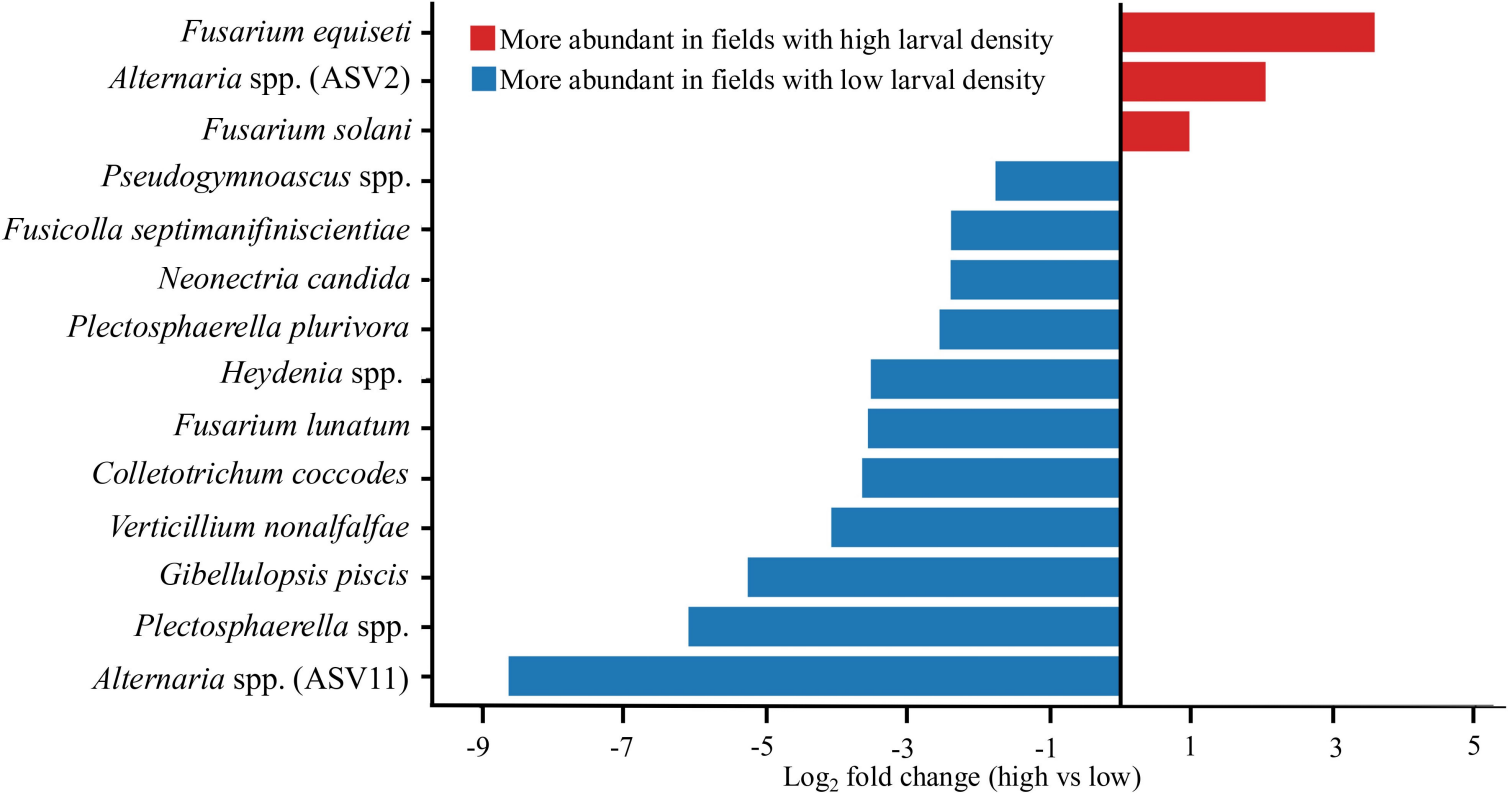
Differentially abundant fungal amplicon sequence variants (ASVs) between fields with high and low *A. karli* larval abundance based on DESeq2 analysis. Bars represent log_2_ fold changes. All shown ASVs met the significance threshold of an adjusted P < 0.05. Red indicates ASVs enriched in high-abundance fields whereas blue indicates ASVs enriched in low-abundance fields.

Fields with low and high larval abundance of *A. karli* harbored distinct fungal communities. Out of 23 core ASVs, 14 ASVs differed between low and high larval abundance fields. Of these, three ASVs were significantly enriched in high-abundance fields, while 11 ASVs were enriched in low-abundance fields. The heatmap of normalized counts revealed a clear block structure that separated samples from fields with low and high abundance of larvae (**Figure 6**), highlighting differences in fungal taxa between fields with high and low larval abundance. Taxa enriched under high larval abundance included *Fusarium*, and *Alternaria*, whereas ASVs enriched in low-abundance fields were dominated by *Gibellulopsis* and *Plectosphaerella*. The horizontal bar plot of log_2_ fold change further confirmed the direction and magnitude of these associations (**Figure 7**). These results highlight taxon-specific shifts in fungal communities in relation to larval abundance.

## 4 Discussion

We identified a diverse assemblage of fungi inhabiting quinoa stems and rhizosphere, encompassing multiple taxa with potential roles as pathogens, endophytes, or saprophytes. Most of the fungal communities associated with quinoa were dominated by phylum Ascomycota. This pattern aligned with a previous research conducted by Cai et al. (2020) who reported that Ascomycota accounted for approximately 60-80% of the total fungal community in the quinoa rhizosphere. Similarly, Ascomycota often are the most prevalent fungi in both stem and rhizosphere communities in other systems (Rungjindamai and Jones, 2024), and a global survey of 235 soils revealed that this phylum dominates soil fungal communities worldwide (Egidi et al., 2019). Moreover, we noted patterns of overlap between several fungal genera we isolated from the quinoa stems and the rhizosphere, suggesting that soil microbiome may serve as a source of stem endophytes, consistent with prior work (Frank et al., 2017; Xiong et al., 2021). For example, *Penicillium* spp., *Fusarium* spp., *Alternaria* spp., and *Plectosphaerella* spp. which have been reported previously as endophytes in quinoa roots (González-Teuber et al., 2017) were dominant in quinoa stems and were also the most abundant fungi in the rhizosphere of quinoa in our surveys. Some of these fungal taxa such as *Penicillium* spp. and *Mortierella* spp. have beneficial roles. For instance, *P. murcianum* has been reported as a symbiotic endophyte that enhanced quinoa salt tolerance by increasing plant height by approximately 22%, photosynthesis by 43%, and stomatal conductance by 38% (González-Teuber et al., 2022). Similarly, *Mortierella alpina* is a plant-growth-promoting fungi and widely used as a biocontrol agent which can antagonize multiple soil-borne pathogens (Ozimek and Hanaka, 2021; Wang et al., 2022; Qiu et al., 2024). In soils naturally suppressive to *Fusarium* wilt disease, *Mortierella* spp. accounted for up to 37% of fungal sequences, highlighting its strong association with pathogen resistance (Xiong et al., 2017). Liu et al. (2025) also reported that *M. alpina* reduced root rot incidence in *Panax notoginseng* Burk (Apiales: Araliaceae) by approximately 85.7% and increased seedling survival to about 82.6%.

The distinct clustering of organically managed quinoa fields (i.e., Alamosa_2023 and Alamosa_2024) from conventionally managed sites highlights the strong influence of management practices in shaping belowground fungal communities. This pattern is consistent with long-term field experiments demonstrating that organic practices restructure soil fungal communities, resulting in higher microbial biomass and distinctly clustered fungal assemblages compared to conventional systems (Hartmann et al., 2015). Organic management has also been shown to foster greater fungal diversity and more complex microbial networks, with increased abundance of keystone taxa (Banerjee et al., 2019). These findings imply that the divergence observed in our quinoa fields reflects a widespread trend that organic management can restructure fungal communities. However, our organic group comprises only two site-years from the same location, so geographic effects cannot be ruled out; additional sites and more sampling are further needed to attribute this separation to management.

Overall, abundance of adult *A. karli* was positively correlated with soil fungal diversity, and we noted specific fungal groups that were enriched in fields with either low or high abundance of adult *A. karli*. This suggests that differences in community composition may contribute to variation in pest pressure. Several genera linked to lower adult densities (e.g., *Cladosporium*, and *Alternaria*) can produce secondary metabolites with insecticidal, antifungal, and nematicidal activities. For instance, *Cladosporium* spp. has been reported as an entomopathogenic fungus causing 71% mortality of the sweetpotato whitefly, *Bemisia tabaci* Gennadius (Hemiptera: Aleyrodidae); 64% of *Metopolophium dirhodum* Walker (Hemiptera: Aphididae); 48% of *Duponchelia fovealis* Zeller (Lepidoptera: Crambidae) and 54% of the old world bollworm, *Helicoverpa armigera* Hübner (Lepidoptera: Noctuidae) (Bahar et al., 2011; Abdelaziz et al., 2018; Amatuzzi et al., 2018; Islam et al., 2019). Similarly, *A. alternata* caused 81% mortality of the green peach aphid, *Myzus persicae* Sulzer (Hemiptera: Aphididae) and 63% mortality of *Lipaphis erysimi* Kaltenbach (Hemiptera: Aphididae) (Paschapur et al., 2022).

In parallel, fields with low larval abundance were enriched in antagonistic endophytes such as *G. piscis* and *Heydenia* spp., which can improve plant vigor and improve plant tolerance to pest injury. For instance, *G. piscis* has been shown to improve host immunity by triggering defense-related pathways such as salicylic acid signaling (Hao et al., 2022). Similarly, *Heydenia alpina* has shown *in vitro* antagonism against plant pathogenic fungi, reducing mycelial growth of *Fusarium oxysporum* by 44% and *A. alternata* by 47% (Yang et al., 2020). While we lack empirical evidence to test this, we speculate that fungal communities associated with low *A. karli* densities (both adults and larvae) were enriched in taxa that could suppress *A. karli* populations through two pathways: (i) direct antagonism because of entomopathogenic properties of fungi (Paschapur et al., 2022) and (ii) improved host vigor that reduces tissue suitability for larval feeding and development (Garzo et al., 2020). Together, these patterns suggest that quinoa fields enriched with these antagonistic and putative entomopathogenic fungi consistently had lower *A. karli* densities because fungal microbiome in these fields not only strengthened quinoa nutrition but also improved fungal antagonism which ultimately reduced adult *A. karli* oviposition and made stem tissues less suitable for larval development.

In contrast, high abundance of *A. karli* adults and larvae was associated with greater relative abundance of pathogenic taxa such as *F. equiseti* and *F. solani*. Several *Fusarium* species such as *F. culmorum*, *F. equiseti*, *F. graminearum*, *F. solani* and *F. oxysporum* are pathogenic to quinoa plants leading to stem and root rots (Fonseca-Guerra et al., 2023; Nebie et al., 2024). The presence of pathogenic fungi can increase plant susceptibility to insect injury. For instance, *F. verticillioides* infection in sugarcane altered volatile emissions, causing caterpillars of sugarcane borer, *Diatraea saccharalis* Fabricius (Lepidoptera: Crambidae) to preferentially colonize *Fusarium*-infected plants and the volatile blend was also less attractive to *Cotesia flavipes*, a parasitoid of the *D. saccharalis* caterpillar (Peñaflor and Bento, 2019; Franco et al., 2021). Enrichment of pathogenic taxa may therefore have contributed to higher *A. karli* abundance by weakening plant resistance and enhancing the nutritional quality of quinoa tissues. A similar mechanism was described in black poplar where the larval growth rate of spongy moth, *Lymantria dispar* L. (Lepidoptera: Erebidae) increased by 33% when fed with rust-infested leaves compared to uninfected leaves. These leaves contained up to 3.8-fold higher mannitol and 2.4-fold more amino acids compared to uninfected controls, demonstrating how fungal infection can enhance nutritional quality for herbivores (Eberl et al., 2020). These results suggest that fields with high relative abundance of pathogenic fungi supported higher *A. karli* densities because pathogen-driven stress can weaken quinoa defenses and make it more susceptible to *A. karli* by altering the tissue quality.

Interestingly, some fungi such as *F. septimanifiniscientiae* were associated with fields with high adult- but low larval- abundance. Members of the *Fusicolla* genus are frequently associated with high concentrations of defensive sulfur compounds, such as glucosinolates (Carpentier et al., 2025). Although glucosinolates themselves are non-volatile, their hydrolysis products (e.g., isothiocyanates) can contribute to volatile emission that mediate plant-insect interactions (Hopkins et al., 2009). One possible explanation is that these volatile emissions may function as semiochemicals (Davis et al., 2013), attracting adult *A. karli* for oviposition while elevated chemical defenses and/or fungal presence subsequently reduce larval performance. The association of some fungal genera (e.g., *Alternaria* spp.*, Plectosphaerella* spp.) with both low and high densities of *A. karli* further suggests that functional roles vary at the species and strain level. For instance, *P. cucumerina* has been isolated as symptomless endophyte from quinoa roots (González-Teuber et al., 2017) but can cause wilt disease in cucumber (Li et al., 2025) and root rot in fennel (Cai et al., 2021). Conversely, in spring barley, *P. cucumerina* acted beneficially by stimulating root growth and increased dry root weight by 56% (Pašakinskienė et al., 2024). These contrasting roles likely explain the patterns observed in our fields. Sites with higher adult *A. karli* may have harbored more pathogenic or stress-inducing *Plectosphaerella* strains, which can weaken host defenses and alter plant cues important for insect colonization. In contrast, sites with lower adult counts may have been dominated by endophytic or growth-promoting strains, which improve root development and plant resilience, making the plants less suitable for infestation. Although several fungal taxa were associated with differences in adult or larval *A. karli* abundance, these patterns should be interpreted as associations rather than evidence of direct causal effects. It is possible that environmental factors we did not measure or account for, such as soil moisture, soil pH, irrigation, nutrient availability, or other edaphic conditions, influenced both fungal community and host plant attractiveness or suitability for *A. karli*. Future studies employing manipulative experiments are required to decouple these environmental drivers from fungal-mediated effects on insect fitness and behavior.

In summary, quinoa stems and rhizosphere hosted diverse but Ascomycota-dominated fungal communities whose composition can play a key role in mediating plant-herbivore interactions. Genera that often include antagonists were more common where pest pressure was low, while pathogenic complexes aligned with higher pest pressure. Management practices such as organic amendments and intercropping can restructure these communities. Therefore, identifying strains that consistently promote plant vigor while suppressing *A. karli* can deliver microbiome-informed, field-ready tactics for sustainable quinoa production.

## Data Availability

The datasets presented in this study can be found in online repositories. The names of the repository/repositories and accession number(s) can be found below: https://www.ncbi.nlm.nih.gov/, PRJNA1437130.
